# ProtPhage: a deep learning framework for phage viral protein identification and functional annotation

**DOI:** 10.1093/bib/bbaf285

**Published:** 2025-06-14

**Authors:** Yuehua Ou, Qiyi Chen, Ningyu Zhong, Zhihua Du

**Affiliations:** College of Computer Science and Software Engineering, Shenzhen University, No. 3688 Nanhai Avenue, Nanshan District, Shenzhen, Guangdong, 518060, China; College of Computer Science and Software Engineering, Shenzhen University, No. 3688 Nanhai Avenue, Nanshan District, Shenzhen, Guangdong, 518060, China; College of Computer Science and Software Engineering, Shenzhen University, No. 3688 Nanhai Avenue, Nanshan District, Shenzhen, Guangdong, 518060, China; College of Computer Science and Software Engineering, Shenzhen University, No. 3688 Nanhai Avenue, Nanshan District, Shenzhen, Guangdong, 518060, China

**Keywords:** phage viral proteins, protein language model, asymmetric loss

## Abstract

Phages, viruses that infect bacteria, offer a promising strategy against antibiotic-resistant pathogens. Phage viral proteins (PVPs) are essential for phage–host interactions, yet their identification and functional annotation remain challenging due to high sequence diversity, limited experimental data, and class imbalance. To address these issues, we propose ProtPhage, a novel framework that leverages the ProtT5 protein language model for richer sequence representation compared to traditional methods. Additionally, ProtPhage incorporates an asymmetric loss function to mitigate class imbalance, significantly improving the prediction of the minority class “minor capsid,” with an F1 score 33.07$\%$ higher than the best existing model. Extensive experiments demonstrate that ProtPhage outperforms current methods across multiple metrics, including accuracy, precision, recall, and F1 score. A case study on the Mycobacterium phage PDRPxv genome further validates its practical utility, while expanded experiments highlight its potential in phage–host prediction. By integrating advanced deep learning techniques, ProtPhage establishes a new standard for PVP identification and annotation, contributing to the broader field of computational phage biology.

## Introduction

Bacteriophages, viruses that specifically infect bacteria, play a crucial role in regulating microbial communities [1]. In recent years, the rapid emergence of antibiotic-resistant pathogens has posed a significant threat to global public health, highlighting the urgent need for alternative antimicrobial strategies [2]. Phage therapy, which utilizes bacteriophages to target and kill bacterial pathogens, has emerged as a promising solution to combat “superbugs” [3, 4]. However, the success of phage therapy heavily depends on accurately identifying the bacterial hosts of phages [5, 6]. Understanding the proteins that constitute the phage viral particle, known as phage viral proteins (PVPs), is essential for elucidating the mechanisms of phage–host interactions and developing novel antibacterial strategies [7]. PVPs are structural components of the phage particle, including the head, tail, and other appendages. These proteins are involved in critical processes such as host recognition, attachment, and the injection of phage genetic material into host cells [8]. Accurate identification and functional annotation of PVPs are not only vital for understanding virus–host dynamics but also crucial for advancing phage therapy and designing novel antibacterial agents [9]. Despite their importance, identifying PVPs remains a challenging task due to the immense diversity of protein sequences, limited experimental data, and the complex functional roles of PVPs.

Traditionally, PVP identification has relied on experimental approaches such as mass spectrometry and protein sequencing [10]. However, these techniques are often labor-intensive, time-consuming, and expensive, making them unsuitable for large-scale analyses. With the advent of high-throughput sequencing technologies [11], the volume of available phage genomic data has increased dramatically. This has created an urgent need for computational methods capable of efficiently and accurately predicting PVPs solely from sequence information [12]. Existing machine learning and deep learning methods, such as PVP-SVM [13], Meta-iPVP [14], PhANNs [15], and DeePVP [16], have made significant progress in PVP identification. Nevertheless, these methods face several limitations, including reliance on predefined feature sets, sparse feature representations, and challenges in handling class imbalance in datasets.

PVP-SVM and DeePVP rely on handcrafted features (e.g. one-hot encoding, physicochemical properties) or similarity-based alignment, which struggle to generalize for low-similarity sequences and imbalanced datasets. PhANNs and Meta-iPVP use traditional machine learning with fixed loss functions, leading to biased predictions for minority classes like “minor capsid.” To address these limitations, we propose ProtPhage, a deep learning-based framework for PVP recognition and functional annotation. ProtPhage integrates ProtT5 [17] embeddings and an asymmetric loss (ASL) function, [18] forming a synergistic solution to the challenges of sequence divergence and class imbalance. ProtT5 embeddings provide rich, context-aware representations that capture evolutionary and functional signals beyond sequence similarity, while the ASL function explicitly emphasizes underrepresented classes during training. This powerful combination enables ProtPhage to deliver robust, generalizable predictions across diverse and imbalanced datasets, addressing critical gaps left by previous methods.

To evaluate the performance of ProtPhage, we conducted extensive experiments on multiple datasets, including both binary and multi-class classification tasks. We further assessed the model’s generalizability across datasets with temporal splits, varying levels of sequence similarity, and differing degrees of class imbalance. Our results demonstrate that ProtPhage outperforms state-of-the-art methods in terms of accuracy, precision, recall, and F1 score, particularly in low-similarity and imbalanced scenarios. Furthermore, we applied ProtPhage to real-world phage genome data, successfully predicting and annotating PVPs. Finally, we explored the potential of the model in the field of phage–host prediction, providing evidence of its utility in advancing research in this area.

## Related work

PVPs play a critical role in the interactions between phages and their bacterial hosts. Accurately identifying PVPs is essential for understanding virus–host dynamics and developing novel antibacterial strategies. Over the years, various computational approaches have been proposed for PVP prediction and classification, each contributing unique innovations and methodologies.

PVP-SVM was introduced by Manavalan *et al*. [13] as a support vector machine-based predictor for PVP identification. This method leverages a carefully selected feature set of 136 descriptors and incorporates a feature selection protocol. It demonstrated high accuracy during leave-one-out cross-validation, outperforming other SVM-based predictors trained on the full feature set.

PVPred-SCM, developed by Charoenkwan *et al*. [19], offers a simpler yet effective scoring card method (SCM) for PVP prediction and analysis. By combining the dipeptide composition with a scoring function, PVPred-SCM computes propensity scores for PVP identification.

PhANNs, proposed by Cantu *et al*. [15], is a machine learning-based tool designed to classify phage open reading frames (ORFs) [20] into structural protein categories or “other” categories. PhANNs achieved high testing accuracy and provides a robust platform for functional annotations of phage proteins.

Meta-iPVP, introduced by Charoenkwan *et al*. [14], represents the first meta-learning approach for PVP prediction. It employs a feature representation scheme that integrates probabilistic information derived from four machine learning algorithms as a function of seven feature encodings. These probabilistic features are used as input to an SVM model [21], with feature selection performed via a genetic algorithm and self-assessment reports.

VirionFinder, developed by Fang and Zhou [22], is a novel algorithm designed to identify complete and partial prokaryotic viral particle proteins. By encoding protein sequences using their biochemical properties and applying deep learning techniques, VirionFinder demonstrated significant advantages in sensitivity, particularly for partial protein sequences.

DeePVP, also developed by Fang *et al*. [16], is a deep learning-based tool capable of distinguishing PVPs from non-PVPs and further classifying them into ten functional categories. DeePVP leverages one-hot encoding and convolutional neural networks (CNNs) [23] to achieve significant improvements in prediction accuracy.

PhaVIP, introduced by Shang *et al*. [24], utilizes chaos game representation [25] and vision transformer models [26] to classify PVPs. By encoding protein sequences into images, the vision transformer effectively learns both local and global sequence features. PhaVIP has demonstrated exceptional performance in classifying PVPs and non-PVPs as well as annotating PVP types, making it a promising tool in the field.

As shown in Table 1, while each method offers unique advantages and innovations, they also have limitations. For instance, as noted by Yunxiao Ren [27], encoding protein sequences with one-hot encoding often results in sparse matrices, leading to the “curse of dimensionality.” Similarly, VirionFinder’s [22] reliance on biochemical properties for encoding protein sequences may not capture the full richness of sequence information, limiting its ability to extract comprehensive features from protein data.

**Table 1:** Summary of the existing PVP classification tools

**Method**	**Year**	**Task**	**Encoding**	**Strengths**	**Weaknesses**
PVP-SVM [13]	2018	Binary classification	K-mer frequency, physicochemical properties	–High accuracy with optimal feature selection –Effective in distinguishing PVPs from non-PVPs	–Relies on feature selection, which may lead to loss of information
PVPred-SCM [19]	2020	Binary classification	K-mer frequency	–Simple and interpretable scoring card method –Effective with dipeptide composition –User-friendly web server for predictions	–Slightly lower accuracy compared to some advanced methods (e.g. PVP-SVM) –Limited to specific types of features
phANNs [15]	2020	Binary classification & Multi-classification	K-mer frequency	–Classifies 10 structural classes + “other” –Large dataset (538K proteins) –ANN with k-mer features	–Low accuracy for minority classes –No explicit sequence context modeling
Meta-iPVP [14]	2020	Binary classification	Probabilistic matrix	–Meta-learning approach with probabilistic features –Integrates 7 feature encodings –Improved accuracy over SVM-based tools	–Small training set (313 PVPs) –Handcrafted features lack biological context –No multi-class classification
VirionFinder [22]	2021	Binary classification	One-hot, physicochemical properties	–Uses sequence and biochemical properties for classification –High sensitivity compared to existing tools	–May struggle with highly divergent proteins –Relies on biochemical properties, which may not capture the full richness of sequence information
DeePVP [16]	2022	Binary classification & Multi-classification	One-hot	–Binary + multi-class classification (10 classes) –Uses one-hot encoding + CNN	–Relies on sparse one-hot encoding –No handling of class imbalance –Limited performance on low-similarity sequences
PhaVIP [24]	2023	Binary classification & Multi-classification	FCGR	–Utilizes Chaos Game Representation for encoding –Employs Vision Transformer for classification –Capable of multi-class classification of PVPs	–Requires high computational resources –Performance may vary on low-similarity sequences

These approaches collectively contribute to advancing PVP prediction and functional understanding of phage proteins. However, there remains room for improvement in capturing complex sequence patterns and addressing limitations such as data sparsity and limited feature representation.

## Method

In this section, we introduce the proposed computational framework for identifying phage virulent proteins (PVPs). The framework is designed to leverage protein language models and neural network classifiers for accurate PVP identification. The hyperparameters and architectural details of the model are shown in Table 2. Below, we provide a detailed explanation of the framework, divided into subsections for clarity.

**Table 2:** Detail construction parameters of ProtPhage

Layer type	Key parameters	Output shape
Input	/	(Batch, 1024) $\rightarrow $ (batch, 1, 1024)
Conv1d	32 filters, kernel=3, stride=1	(Batch, 32, 1022)
BatchNorm1d	num_features=32	(Batch, 32, 1022)
MaxPool1d	kernel=3, stride=2	(Batch, 32, 510)
Flatten	/	(Batch, 16320)
LazyLinear	out_features=64	(Batch, 64)
BatchNorm1d	num_features=64	(Batch, 64)
Linear	in_features=64, out_eatures=2	(Batch, 2)
Asymmetric loss (ASL) Parameters		
$\gamma _{pos}$	0	/
$\gamma _{neg}$	4	/

### Overview

**Figure 1:**
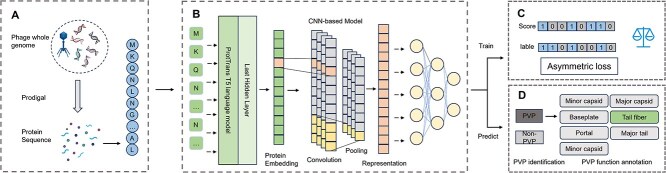
Workflow of the ProtPhage. (A) Translation: processing genomic sequences (B) Model framework, including feature extractor and classifiers. (C) Training with ASL (D) PVP indentification and function annotation.

The proposed pipeline integrates biological data processing with advanced computational techniques to identify PVPs from phage genomic sequences. The workflow begins by translating genomic sequences into protein sequences, which are then passed through a pre-trained protein language model (ProtT5) for feature extraction. The extracted embeddings are subsequently classified using a multi-class neural network, and detailed functional annotations are provided for the predicted PVPs, Fig. 1 illustrates the overall architecture of the framework.

### Translation: processing genomic sequences

As shown in Fig. 1A, ORF identification: using bioinformatics tools, such as Prodigal [28] or GeneMark [29], ORFs are identified from the input phage genomic sequences. Each ORF is translated into its corresponding amino acid sequence using the standard genetic code. This step produces strings of amino acid residues (e.g. “MKQN...”), which are used as inputs for feature extraction. This process ensures that the downstream model operates on protein-level data, which is more relevant for functional prediction tasks. The output of this stage is a collection of protein sequences, each corresponding to an ORF in the input genomes.

### Feature extraction: embedding protein sequences with ProtT5

The protein sequences generated in the previous step are transformed into high-dimensional, context-aware feature representations using the ProtT5 language model. ProtT5, a transformer-based model pre-trained on large protein sequence datasets, captures both local and global dependencies within sequences, making it highly effective for protein classification tasks. It is worth mentioning that our study is the first to apply protein language models to PVP identification tasks.

As shown in Fig. 1B, the feature extraction process involves several key steps. First, each protein sequence is tokenized into its individual amino acid residues. For instance, the sequence “MKQN” is split into the tokens “M,” “K,” “Q,” and “N.” These tokenized sequences are then fed into ProtT5’s encoder, which incorporates essential components such as multi-head attention mechanisms and feedforward layers. This architecture enables the model to capture complex relationships between amino acid residues, generating contextual embeddings for each residue.

These embeddings encapsulate biologically relevant information, including residue interactions and conserved motifs. From the last layer of the pre-trained model, we extract the average embedding vector, resulting in a 1024-dimensional feature vector for each protein sequence. These embeddings are then used as input for the classifier, allowing the model to leverage both sequence-level and residue-level information for effective classification.

### Prediction and functional annotation

In the classifier, we employed a convolutional neural network (CNN) to process the obtained embeddings. The architecture of the neural network consists of one-dimensional convolutional layers, batch normalization layers, and pooling layers. Initially, the convolutional layer performs upsampling on the embeddings, resulting in multi-channel feature representations. Following this, the batch normalization layer normalizes these features, producing standardized representations that enhance model stability and performance. The pooling layer then applies pooling operations to increase the receptive field of the network, which helps to mitigate the impact of noise on the model’s predictions. Subsequently, we utilized a fully connected neural network [30] to introduce non-linear transformations to the features processed by the CNN. This final stage culminates in the prediction of protein classifications.

As shown in Fig. 1D, to obtain more detailed classification information, we conducted further functional annotations on proteins predicted as PVP. This annotation process categorizes the proteins into several distinct classes: “portal,” “major capsid,” “minor capsid,” “major tail,” “minor tail,” “baseplate,” and “tail fiber.” This additional analysis enriches our understanding of these proteins’ roles and characteristics within biological systems.

### Training with ASL loss function

In our model, we employed the ASL function to mitigate the challenges posed by class imbalance during training, as shown in Fig. 1C. Class imbalance is a prevalent issue in classification tasks, where some classes are significantly underrepresented compared to others. This imbalance can lead to biased predictions and a decline in overall model performance.

The ASL function is specifically designed to address this issue by applying different weights to positive and negative samples. The core idea is to increase the penalty for misclassifying minority class instances while reducing the penalty for misclassifying majority class instances. The ASL loss function is calculated as follows:


(1) \begin{align*} & \log_{i}=\log\left(\frac{\exp(x_{i})}{\Sigma_{j}\exp(x_{j})}\right) \end{align*}

(2) \begin{align*} & x\operatorname{spos}=e^{\log_{i}} \cdot y_{i} \end{align*}

(3) \begin{align*} & x\operatorname{sneg}=\left(1-e^{\text{Log} _{i}}\right) \cdot\left(1-y_{i}\right) \end{align*}

(4) \begin{align*} \text{asymmetricWeight}_{i}& =\nonumber\\ & (1-\text{xspos}- \text{xsneg})^{\gamma_{p o s} \cdot y_{i}+\gamma_{n e g} \cdot\left(1-y_{i}\right)} \end{align*}

(5) \begin{align*} & \operatorname{Loss}_{i}=-\sum_{j} y_{i j} \cdot \log_{i} \cdot \text{ asymmetricWeight}_{i} \end{align*}

(6) \begin{align*} & \operatorname{Loss}_{i}=\frac{1}{N} \sum_{i} \operatorname{Loss}_{i} \end{align*}

where ${Log}_{i}$ represents the Logsoftmax function, ${x}_{i}$ represents the original prediction score of the i class, ${x}_{j}$ represents the original prediction score of the j class, ${xspos}$ represents the prediction probability of the virions protein prediction model for the target class, ${xsneg}$ represents the complement of the prediction probability of the virion-protein prediction model for non-target classes, ${y}_{i}$ represents the real label of the ${i}{th}$, ${asymmetricWeight}_{i}$ represents the asymmetric weight, $\gamma _{pos}$ represents the weight attenuation factor controlling the positive sample, $\gamma _{neg}$ represents the weight attenuation factor controlling the negative sample.

By adjusting the asymmetric weight parameters $\gamma _{pos}$ and $\gamma _{neg}$ of positive and negative samples in the loss function, the model can be better trained for the class with fewer samples. Our model not only mitigated the adverse effects of class imbalance but also improved its sensitivity to underrepresented classes. This approach allowed for a more balanced learning process, ensuring that the model does not become overly biased toward the majority class.

## Experiments

The ProtPhage framework tackles two tasks: binary classification to distinguish between PVP and non-PVP sequences, and multi-class classification to identify seven specific types of PVPs. To ensure effective training, we treated these tasks separately.

### Data collection

In our study, we used data similar to that in the PhaVIP. To update the viral protein database for PVP classification, we downloaded the latest annotations from RefSeq (December 2022). Following third-party review guidelines, we first identified proteins belonging to bacteriophages. Structural protein sequences were extracted using keywords such as “portal,” “capsid,” “tail,” “fiber,” “tape measure,” “baseplate,” and “structural.” For non-structural proteins, we searched for enzyme-related annotations, such as those ending in “ase.” Additionally, keywords like “transcription,” “holin,” “lysin,” and “regulator” were used to construct the non-PVP dataset. As a result, our dataset consisted of 35 213 PVP sequences and 46 883 non-PVP sequences.

When constructing the training, validation, and test sets, we divided the data differently based on the specific tasks. For the binary classification task, all proteins were used for data splitting. For the multi-class classification task, we built the dataset using proteins annotated with “portal,” “major capsid,” “minor capsid,” “major tail,” “minor tail,” “baseplate,” and “tail fiber.”

To comprehensively evaluate the model’s performance, we have meticulously divided the aforementioned dataset based on various conditions.

Split by time: Splitting the training and test sets based on time is a widely used method, as it mimics the real-world scenario of discovering unknown PVPs using known PVPs. In this dataset, proteins released before December 2020 were used to construct the training set, while proteins released after December 2020 were used to construct the test set. Ultimately, 27 704 PVP sequences and 36 778 non-PVP sequences were included in the training set, while 7509 PVP sequences and 10 103 non-PVP sequences were included in the test set. To balance the dataset for the binary classification task, we randomly sampled non-PVP sequences to match the number of PVP sequences. For the multi-class classification task, the original data distribution was preserved.

Split by similarity: To evaluate the model’s performance under varying levels of sequence similarity, we constructed datasets with different sequence similarity thresholds between protein sequences in the test set and the training set. Specifically, we applied all-against-all BLASTP searches to our PVP dataset and calculated the product of pairwise sequence identity and alignment coverage, where alignment coverage was defined as the ratio of alignment length to the query sequence length. Based on this, we specified a maximum similarity threshold between the training and test sets to create distinct datasets. We selected thresholds of 0.4, 0.5, 0.6, 0.7, 0.8, and 0.9, and used stratified sampling to split the training and test sets accordingly.

Split by imbalance ratio: In order to assess the model’s performance under varying balance factors (numbers of non-PVP sequences / numbers of PVP sequences), we constructed datasets with different balance factors for the binary classification task. Specifically, we randomly sampled from 35 213 PVP sequences and 46 883 non-PVP sequences according to various balance factors, thereby creating training and testing sets with the same balance factors. In this study, we selected 1, 3, 5, 7, and 9 as the balance factors.

### Visualized the features

**Figure 2:**
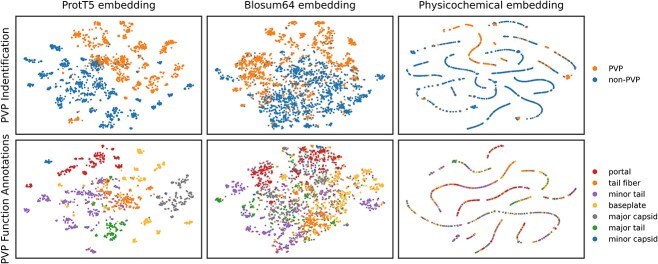
t-SNE visualization of three types of feature embedding.

To evaluate the feasibility of using protein language models (PLMs) for PVP recognition and functional prediction tasks, we compared feature representations encoded by ProtT5 embeddings, evolutionary information-based Blosum64 [31], and physicochemical embeddings using t-SNE visualization. The results, summarized in Fig. 2, showcase the discriminative ability of these features in binary and multi-class classification tasks on the split-by-time dataset.

In the PVP recognition task, ProtT5 embeddings demonstrated superior separability between PVP and non-PVP sequences compared to Blosum64 and physicochemical embeddings. ProtT5 formed distinct clusters for PVP and non-PVP sequences, effectively capturing key viral protein properties, such as functional characteristics. In contrast, Blosum64 embeddings showed moderate overlap between classes, while physicochemical embeddings exhibited scattered patterns with poor clustering. These results highlight ProtT5’s ability to capture biologically relevant features for distinguishing PVPs.

For the functional prediction task, ProtT5 embeddings again outperformed the other methods. Functional categories such as “portal,” “tail fiber,” and “major capsid” formed well-defined clusters, indicating that ProtT5 effectively captures relationships among proteins with similar functions. While Blosum64 embeddings showed partial clustering, the boundaries between categories were less distinct. Physicochemical embeddings performed the worst, showing widely dispersed categories with no clear separability.

These findings demonstrate that PLMs, particularly ProtT5, excel in capturing functional properties of viral proteins, surpassing traditional approaches like Blosum64 and physicochemical embeddings. ProtT5’s superior separability and clustering in both PVP recognition and functional prediction tasks highlight its potential for accurate PVP identification and functional annotation.

### Optimization of ASL hyperparameters

The selection of appropriate values for $\gamma _{pos}$ and $\gamma _{neg}$ in the ASL function is crucial for addressing class imbalance effectively. To determine the optimal parameters, we conducted a systematic evaluation of different ($\gamma _{pos}$, $\gamma _{neg}$) combinations and their impact on model performance across all PVP categories.

**Figure 3:**
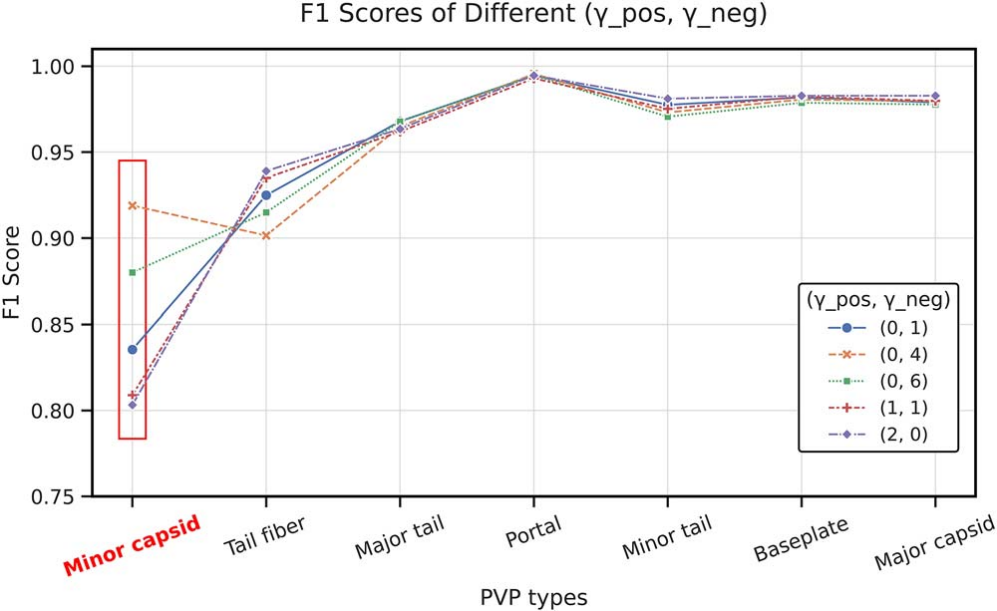
ASL hyperparameters selection experimental results.

We tested five configurations on the dataset split by time: (0,1), (0,4), (0,6), (1,1), and (2,0), with results shown in Fig. 3. The evaluation shows that the overall performance of ($\gamma _{pos}$, $\gamma _{neg}$) = (0,4) is the best, especially for the minority class “minor capsid”. Compared with the setting of ($\gamma _{pos}$, $\gamma _{neg}$) = (1,1), it increases the F1 score from 0.8089 to 0.9189. This 11% improvement demonstrates the importance of proper parameter selection for minority classes.

The analysis showed that higher $\gamma _{neg}$ values (4 or 6) significantly enhanced the model’s sensitivity to underrepresented classes by increasing the penalty for misclassifying their samples. In contrast, configurations with $\gamma _{pos}$ > 0 [e.g. (1,1) and (2,0)] tended to favor majority classes at the expense of minority class performance. The (0,4) configuration provided the best balance, improving recall for minority classes without substantially compromising precision for majority classes.

### Performance on the benchmark dataset split by time

To evaluate the temporal generalization capability of the models, we trained ProtPhage on the split-by-time dataset and compared its performance with several state-of-the-art methods, including phANNs, PhaVIP, VirionFinder, and others. For the baseline methods, we used the pre-trained models provided by their respective authors and applied the recommended parameters during testing. The results of all methods are summarized in Table 3. ProtPhage outperformed all other approaches, achieving an accuracy of 0.9717, a precision of 0.9528, and an F1 score of 0.9673 on the test set. Notably, ProtPhage demonstrated the ability to accurately predict future PVP sequences, highlighting its strong temporal generalization capability. These findings underscore the potential of ProtPhage for practical applications in real-world scenarios.

**Table 3:** Benchmarking PVP identification of different methods using split by time dataset

Method	ACC	Precision	Recall	F1
phANNs	0.905	0.8917	0.8845	0.8881
DeePVP	0.7892	0.9862	0.5127	0.6746
VirionFinder	0.6196	**0.9966**	0.118	0.211
pvp_svm	0.7446	0.8227	0.5111	0.6305
Meta-iPVP	0.8689	0.8537	0.8617	0.858
PVPred-SCM	0.8016	0.8314	0.7526	0.79
PhaVIP	0.925	0.8945	0.9343	0.914
ProtPhage	**0.9717**	0.9528	**0.9823**	**0.9673**

***Bold** values indicate the highest score achieved for each metric.*

Next, we evaluated the performance of ProtPhage on the multi-class classification task. Among the existing methods, only phANNs, PhaVIP, and DeePVP are capable of providing more detailed annotations for PVPs. However, the label definitions in the original designs of phANNs and DeePVP differ from those in our model, and only phANNs allows retraining on the multi-class classification dataset. Therefore, we retrained phANNs on the dataset and tested PhaVIP without modification. The results are summarized in Table 4. ProtPhage outperformed the other two models, achieving an overall F1 score of 0.9686.

**Table 4:** Benchmarking PVP function annotations of different methods using split by time dataset

Method	ACC	Precision	Recall	F1	MCC
phANNs	0.927	0.9287	0.927	0.9277	0.9081
PhaVIP	0.9004	0.9014	0.9004	0.9003	0.8753
ProtPhage	**0.9679**	**0.9707**	**0.9679**	**0.9686**	**0.9600**

Bold values indicate the highest score achieved for each metric.

**Figure 4:**
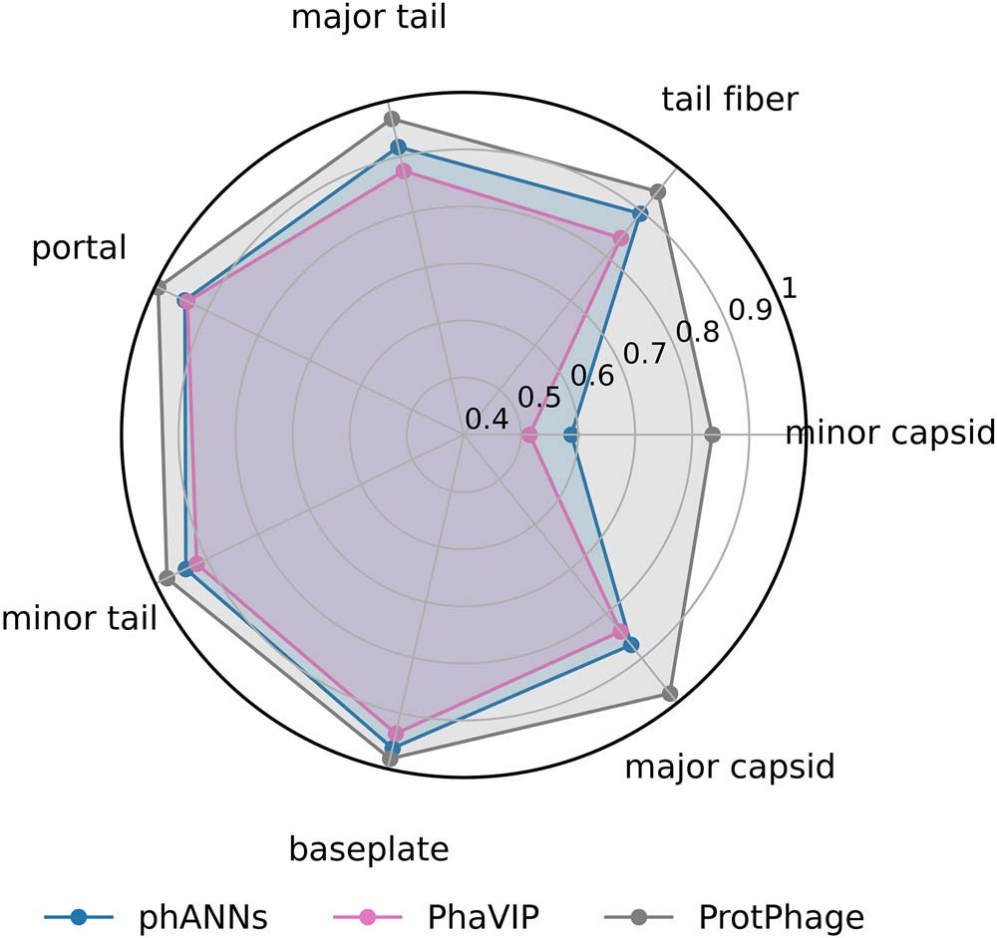
Radar plot of model F1 score across seven categories.

The F1 scores for individual categories are shown in Fig. 4. The results indicate that the multi-class classification task is substantially more challenging than the binary classification task. This could be attributed to the smaller amount of training data available for each category and the class imbalance inherent in the dataset. Among these categories, minor capsid presents the most significant challenge due to its severely imbalanced sample size (only 252 samples compared to 1819 for baseplate and 2565 for minor tail). The inherent class imbalance in the dataset makes it difficult for models to achieve high performance on minority classes.

ProtPhage, however, demonstrates a clear advantage in handling this imbalance, especially for the minor capsid category. By incorporating ASL during training, ProtPhage effectively adjusts the loss function to focus more on minority classes, mitigating the impact of the imbalanced data distribution. ProtPhage achieves a substantially higher F1 score for the minor capsid class compared to phANNs and PhaVIP. ProtPhage achieved an F1 score of 0.9189 for the minor capsid category, significantly outperforming phANNs (0.5882) and PhaVIP (0.5151).

### Performance on the benchmark dataset split by similarity

Existing PVP recognition tools often rely on sequence similarity for making predictions. However, such approaches typically struggle to predict and annotate highly divergent proteins. To evaluate the performance of models under varying levels of sequence divergence, we used the product of Identity and Coverage as a similarity metric to control the maximum similarity between the training and test sets. Using this metric, we generated six datasets with different levels of sequence similarity for both binary and multi-class classification tasks. The performance of various methods was then evaluated on these datasets.

**Figure 5:**
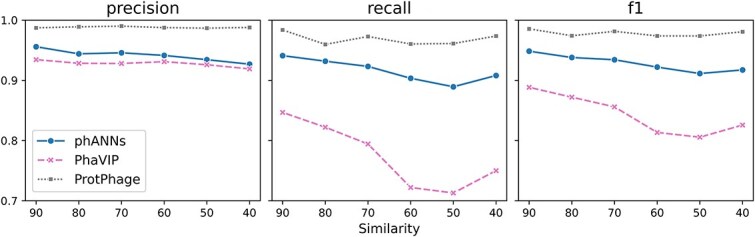
Benchmarking pvp identification of different methods using split by similarity dataset.

The results, shown in Fig. 5, indicate that in the binary classification task, the F1 scores of all methods decrease as the similarity between the training and test sets diminishes. However, ProtPhage consistently achieved the highest F1 scores across all similarity datasets. Notably, as sequence similarity decreases, the performance gap between ProtPhage and other methods widens. This highlights the increasing importance of leveraging the ProtT5 pre-trained model to capture functional homology in low-similarity regions, where traditional sequence similarity-based methods often fail.

Similarly, in the multi-class classification task, ProtPhage demonstrated the same trend. The results are summarized in Table 5. ProtPhage achieved the best performance across all datasets with different levels of similarity. Moreover, the performance of ProtPhage remained stable and did not exhibit significant fluctuations as sequence similarity decreased. The clear performance gap between ProtPhage and other methods underscores the competitive advantage of our model in handling a wide range of sequence similarities for both binary and multi-class classification tasks.

**Table 5:** Benchmarking pvp function annotations of different methods using split by similarity dataset

Similarity	Method	Acc	Precision	Recall	F1	MCC
	phANNs	0.519	0.5451	0.519	0.5076	0.4142
40	PhaVIP	0.4648	0.4787	0.4648	0.4559	0.3395
	**ProtPhage**	**0.8027**	**0.8129**	**0.8027**	**0.7985**	**0.7573**
	phANNs	0.5328	0.5271	0.5328	0.5168	0.4217
50	PhaVIP	0.498	0.5139	0.498	0.5015	0.3902
	**ProtPhage**	**0.8009**	**0.8186**	**0.8009**	**0.7670**	**0.7570**
	phANNs	0.6385	0.6407	0.6385	0.6369	0.5585
60	PhaVIP	0.5294	0.5373	0.5294	0.5293	0.4267
	**ProtPhage**	**0.8298**	**0.8359**	**0.8298**	**0.8296**	**0.7922**
	phANNs	0.6439	0.6452	0.6439	0.6312	0.5616
70	PhaVIP	0.5793	0.5853	0.5793	0.5809	0.4847
	**ProtPhage**	**0.8211**	**0.8142**	**0.8211**	**0.8144**	**0.7804**
	phANNs	0.6941	0.695	0.6941	0.69	0.6238
80	PhaVIP	0.578	0.5813	0.578	0.5767	0.4816
	**ProtPhage**	**0.8426**	**0.8417**	**0.8426**	**0.8391**	**0.8061**
	phANNs	0.7518	0.7604	0.7518	0.7538	0.6963
90	PhaVIP	0.6818	0.6728	0.6818	0.6755	0.606
	**ProtPhage**	**0.8707**	**0.8713**	**0.8707**	**0.8678**	**0.8407**

To further investigate the superior performance of ProtPhage in low-similarity scenarios, we conducted comparative experiments by replacing ProtT5 embeddings with BLOSUM64 and physicochemical features. As shown in Supplementary Table S1, ProtT5 consistently outperformed traditional features across all similarity levels—achieving an F1 score of 0.9811 at 40% similarity for binary classification, compared to 0.8669 (BLOSUM64) and 0.6721 (physicochemical). This demonstrates that ProtT5’s ability to capture functional homology, such as conserved motifs and structural domains, is key to its robustness, while traditional features degrade sharply with sequence divergence.

### Performance on the benchmark dataset split by imbalance ratio

Deep learning-based predictive models are influenced by the distribution of training data. In real-world scenarios, data distributions are often imbalanced, which can result in models being biased toward majority classes, leading to suboptimal predictions for minority classes. To address this issue, ProtPhage incorporates the ASL function during training, which adjusts the weight decay factor for positive and negative samples to mitigate the impact of class imbalance.

**Figure 6:**
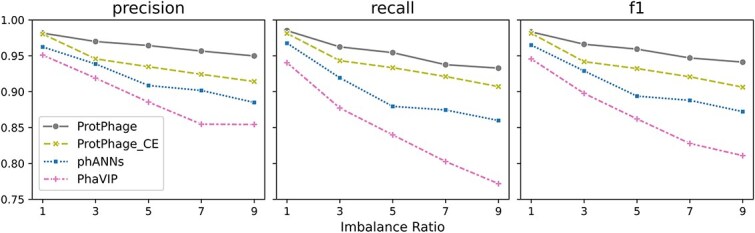
Benchmarking PVP identification of different methods using split by imbalance ratio dataset.

To rigorously evaluate the impact of our proposed ASL function, we constructed five datasets with increasingly imbalanced distributions for the binary classification task and conducted an ablation study comparing ProtPhage trained with ASL against ProtPhage trained with standard cross-entropy (CE) loss, alongside baseline methods (phANNs, PhaVIP). Each method was evaluated on these datasets, and the results are summarized in Fig. 6.

As class imbalance intensifies, all methods exhibit progressive performance degradation, with particularly pronounced declines in minority-class recall. The standard CE-loss variant of ProtPhage mirrors this trend, demonstrating the inherent bias of conventional loss functions toward majority classes. In contrast, ProtPhage with ASL maintains consistently robust performance across all imbalance levels, showing markedly smaller fluctuations between precision and recall compared to both its CE-loss counterpart and baseline methods. This stability confirms ASL’s ability to dynamically compensate for distribution skew while preserving discriminative power across all classes—a critical advantage for real-world PVP prediction where extreme imbalance is commonplace.

### Case study: annotating proteins on the mycobacteriophage PDRPxv genome

In this case study, we utilized ProtPhage to annotate the proteins translated from Mycobacteriophage PDRPxv, which is deemed a potential therapeutic candidate for treating mycobacterial infections. As per the research conducted by Avni Sinha *et al*. [10], a total of 107 protein sequences were translated from the PDRPxv genome. They identified 12 PVPs using mass spectrometry and 12 non-PVPs using the BLAST tool. Since PDRPxv was not present in the training set, we were able to directly apply the trained model to make predictions on it, thereby evaluating the model’s predictive performance in real-world scenarios.

We fed the 107 protein sequences into the model, starting with a binary classification task, namely PVP identification. The results are presented in Table 6. ProtPhage successfully identified all 12 PVPs, outperforming other comparative methods, thereby validating its reliability in PVP recognition. Additionally, we assessed the model’s performance in multi-class classification tasks. As shown in Supplementary Table S2, the findings reveal that out of the 12 predicted PVP sequences, ProtPhage accurately annotated 8 with high confidence scores (>0.997). For the remaining four sequences whose categories fall outside the model’s 7 predefined types, ProtPhage appropriately assigned moderate-to-low confidence scores (ranging from 0.3255 to 0.6917) to its predictions, demonstrating its ability to distinguish between in-class and out-of-class predictions. This case study substantiates ProtPhage’s potential for precise prediction when applied to practical situations, while also showing appropriate caution when encountering protein types beyond its training scope.

**Table 6:** The performance of classifying proteins in mycobacteriophage PDRPxv genome

Method	PVP identification	PVP function annotation
phANNs	7/12	6/7
DeePVP	9/12	8/9
PhaVIP	11/12	7/11
**ProtPhage**	**12/12**	**8/12**

Bold values indicate the results from the ProtPhage model.

### Using classified proteins for the phage host prediction

In recent years, the rapid emergence of antibiotic-resistant pathogens has made phage therapy a promising alternative to antibiotics for combating “superbugs” [32]. Consequently, accurately identifying the hosts of phages is critical for utilizing phages to treat bacterial infections. In this study, we focused on two common pathogens: *Escherichia coli* and *Pseudomonas aeruginosa*.

**Figure 7:**
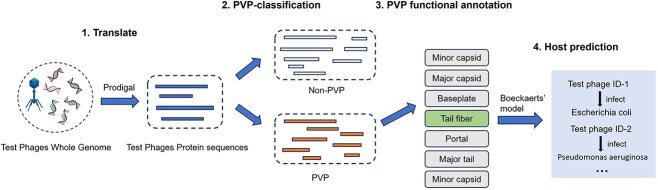
The pipeline of using predicted PVP sequence for host prediction.

For phage–host predictions, existing machine learning methods predominantly rely on receptor-binding proteins, which belong to the PVP family. As shown in Fig. 7, we first collected the whole genome data of four phages: Enterobacteria phage P4 and Enterobacteria phage BA14, which infect *E. coli*, and Pseudomonas phage H70 and Pseudomonas phage vB_PaeS_PAO1_Ab30, which infect *P. aeruginosa*. These phages are well-studied and widely used in research. Using the Prodigal tool, we translated the genomic data into corresponding phage proteins. Next, we applied our trained ProtPhage to predict PVPs and non-PVPs from the phage protein datasets. For the predicted PVPs, we further performed functional annotations.

Following the method described by Boeckaerts *et al*. [33], we filtered the predicted PVPs to identify proteins annotated as tail fibers, which were then used as input for Boeckaerts’ host prediction model.

The results, summarized in Table 7, demonstrate the effectiveness of this approach. For example, Enterobacteria phage P4 contains 15 proteins, of which ProtPhage identified three as PVPs. Among these, one protein was annotated as a tail fiber, which successfully led to the correct host prediction. Similar results were observed for the other three phages. These findings suggest that accurate PVP predictions can significantly narrow the scope for phage–host prediction models, thereby improving prediction accuracy. This underscores the potential of ProtPhage in advancing the field of phage–host prediction.

**Table 7:** Summary of host prediction for *E. coli* and *P. aeruginosa* phages using predicted PVPs

Method	Number of predicted PVP / Sum of proteins	Number of predicted tail fiber	Correct?
Enterobacteria phage P4	3/15	1	$\surd $
Enterobacteria phage BA14	18/44	6	$\surd $
Pseudomonas phage H70	27/56	7	$\surd $
Pseudomonas phage H70	26/55	6	$\surd $

## Conclusion and discussion

In this study, we introduced ProtPhage, a novel deep learning framework for PVP identification and functional annotation. By leveraging ProtT5 embeddings, our model effectively captures rich sequence information, enabling more accurate classification of PVPs compared to existing approaches. Furthermore, to address the inherent class imbalance in PVP datasets, we integrated an ASL function, which significantly improved the model’s ability to generalize across diverse datasets.

Through extensive experiments, ProtPhage demonstrated state-of-the-art performance in both binary classification (distinguishing PVPs from non-PVPs) and multi-class classification (functional annotation of PVPs). Our results indicate that ProtPhage consistently outperforms existing methods, including phANNs, PhaVIP, and DeePVP, across multiple evaluation settings. Particularly, ProtPhage exhibits strong generalization capabilities when handling low-similarity sequences, which is crucial for identifying novel viral proteins from newly discovered phage genomes. The model also maintained high accuracy under varying levels of class imbalance, showcasing its robustness in real-world datasets where certain PVP categories are underrepresented.

To validate the practical utility of ProtPhage, we conducted a case study on the mycobacteriophage PDRPxv genome, demonstrating its ability to correctly identify and functionally annotate PVPs. Additionally, we explored how ProtPhage’s predictions could be leveraged for phage–host prediction, revealing its broader impact on bacteriophage therapy and antibacterial strategies. By accurately identifying tail fiber proteins—a key determinant of phage-host specificity—ProtPhage can facilitate more effective host prediction models, thereby aiding in the selection of therapeutic phages against antibiotic-resistant bacteria.

While ProtPhage demonstrates promising performance, several limitations and improvement directions should be noted. First, the current training data may reflect biases in phage research focus, with overrepresentation of well-studied families. Future versions could address this through active collaborations with phage biobanks to sequence underrepresented families and by incorporating adversarial training techniques to decouple taxonomic bias from feature learning. Second, although ProtPhage currently covers seven major PVP categories (portal, major/minor capsid, major/minor tail, baseplate, and tail fiber), many newly discovered types remain unclassified. ProtPhage’s modular architecture enables straightforward adaptation to newly discovered PVP types through incremental learning—where only the classifier head requires retraining on new categories while preserving the pre-trained ProtT5 embeddings. A community-driven annotation platform could accelerate this process by aggregating experimentally validated PVPs. Third, while ProtT5 embeddings effectively capture sequence patterns, they lack explicit structural and biochemical feature integration. Combining such features with ProtT5 embeddings may provide deeper biological insights into viral protein functions. Fourth, although the model handles moderate sequence divergence well, extremely divergent sequences (<30% similarity) still pose challenges. Additional fine-tuning on newly annotated datasets could further improve its adaptability to emerging phage sequences. Finally, current applications are limited to isolated phage genomes rather than complex communities. Applying ProtPhage to metagenomic datasets could open new avenues in viral ecology and microbiome research, enabling large-scale discovery and functional characterization of phage proteins in complex microbial environments.

Overall, ProtPhage represents a significant step forward in computational phage protein analysis, offering a powerful tool for phage therapy development, antibiotic resistance mitigation, and fundamental virology research. As the volume of phage genomic data continues to expand, we anticipate that ProtPhage will play a crucial role in advancing our understanding of phage biology and accelerating the discovery of novel antibacterial agents.

**Key Points**

- ProtPhage framework: ProtPhage is a deep learning framework for identifying and annotating phage viral proteins (PVPs) using the ProtT5 model.
- Class imbalance handling: it employs an asymmetric loss function to improve prediction accuracy for minority classes, especially “minor capsid”.
- Performance excellence: ProtPhage outperforms existing methods in accuracy, precision, recall, and F1 score, setting a new standard for PVP identification.
- Practical application: a case study on mycobacterium phage PDRPxv demonstrates ProtPhage’s effectiveness in real-world PVP prediction and annotation.
- Phage therapy impact: by enhancing PVP identification, ProtPhage contributes to the development of effective phage therapies against antibiotic-resistant bacteria.

## Supplementary Material

ProtPhage_Supplementary_Information_bbaf285

## Data Availability

The datasets and codes are available at https://github.com/YuehuaOu/ProtPhage.
