# Supplementary material for: ProtPhage: a deep learning framework for phage viral protein identification and functional annotation
Source: Brief Bioinform. 2025 Jun 14;26(3):bbaf285. doi: 10.1093/bib/bbaf285 (PMC12165830; doi:10.1093/bib/bbaf285)
Supplement: ProtPhage_Supplementary_Information_bbaf285 [file protphage_supplementary_information_bbaf285.docx]

Supplementary Information

for the manuscript entitled “ProtPhage: A Deep Learning Framework for Phage

Viral Protein Identification and Functional Annotation”

**Table S1.** Performance comparison of ProtPhage using ProtT5, BLOSUM62 and physicochemical embeddings on split by similarity dataset. (a) PVP identification (b) PVP function annotations

| Similarity | Method | Acc | Precision | Recall | F1 | Mcc |
| --- | --- | --- | --- | --- | --- | --- |
| 40 | ProtPhage-ProtT5 | 0.9813 | 0.9846 | 0.9778 | 0.9811 | 0.9625 |
|  | ProtPhage-Blosum64 | 0.8719 | 0.9022 | 0.8343 | 0.8669 | 0.7459 |
|  | ProtPhage-Physicochemical | 0.6046 | 0.5740 | 0.8105 | 0.6721 | 0.2296 |
| 50 | ProtPhage-ProtT5 | 0.9690 | 0.9865 | 0.9510 | 0.9684 | 0.9386 |
|  | ProtPhage-Blosum64 | 0.8419 | 0.9129 | 0.7558 | 0.8270 | 0.6942 |
|  | ProtPhage-Physicochemical | 0.6194 | 0.5929 | 0.7618 | 0.6668 | 0.2493 |
| 60 | ProtPhage-ProtT5 | 0.9741 | 0.9858 | 0.9621 | 0.9738 | 0.9485 |
|  | ProtPhage-Blosum64 | 0.8772 | 0.9167 | 0.8300 | 0.8712 | 0.7579 |
|  | ProtPhage-Physicochemical | 0.7714 | 0.7096 | 0.9194 | 0.8009 | 0.5683 |
| 70 | ProtPhage-ProtT5 | 0.9812 | 0.9826 | 0.9798 | 0.9812 | 0.9625 |
|  | ProtPhage-Blosum64 | 0.8813 | 0.9340 | 0.8207 | 0.8737 | 0.7684 |
|  | ProtPhage-Physicochemical | 0.8198 | 0.7842 | 0.8826 | 0.8305 | 0.6447 |
| 80 | ProtPhage-ProtT5 | 0.9725 | 0.9868 | 0.9579 | 0.9721 | 0.9455 |
|  | ProtPhage-Blosum64 | 0.8715 | 0.9227 | 0.8108 | 0.8631 | 0.7485 |
|  | ProtPhage-Physicochemical | 0.6236 | 0.5911 | 0.8006 | 0.6801 | 0.2643 |
| 90 | ProtPhage-ProtT5 | 0.9819 | 0.9886 | 0.9751 | 0.9819 | 0.9640 |
|  | ProtPhage-Blosum64 | 0.8900 | 0.9253 | 0.8487 | 0.8853 | 0.7828 |
|  | ProtPhage-Physicochemical | 0.7553 | 0.6990 | 0.8966 | 0.7856 | 0.5323 |

1. PVP identification

| Similarity | Method | Acc | Precision | Recall | F1 | Mcc |
| --- | --- | --- | --- | --- | --- | --- |
| 40 | ProtPhage-ProtT5 | 0.8026 | 0.8129 | 0.8027 | 0.7985 | 0.7573 |
|  | ProtPhage-Blosum64 | 0.5879 | 0.5885 | 0.5879 | 0.5545 | 0.4953 |
|  | ProtPhage-Physicochemical | 0.3521 | 0.2373 | 0.3521 | 0.2808 | 0.1702 |
| 50 | ProtPhage-ProtT5 | 0.8009 | 0.8186 | 0.8009 | 0.7970 | 0.7570 |
|  | ProtPhage-Blosum64 | 0.6073 | 0.5976 | 0.6073 | 0.5959 | 0.5135 |
|  | ProtPhage-Physicochemical | 0.2901 | 0.1823 | 0.2900 | 0.2232 | 0.0994 |
| 60 | ProtPhage-ProtT5 | 0.8298 | 0.8359 | 0.8298 | 0.8296 | 0.7922 |
|  | ProtPhage-Blosum64 | 0.6337 | 0.6197 | 0.6337 | 0.6175 | 0.5468 |
|  | ProtPhage-Physicochemical | 0.3239 | 0.1904 | 0.3239 | 0.2357 | 0.1328 |
| 70 | ProtPhage-ProtT5 | 0.8211 | 0.8142 | 0.8211 | 0.8144 | 0.7804 |
|  | ProtPhage-Blosum64 | 0.6633 | 0.6468 | 0.6633 | 0.6468 | 0.5864 |
|  | ProtPhage-Physicochemical | 0.3603 | 0.2891 | 0.3603 | 0.3125 | 0.2001 |
| 80 | ProtPhage-ProtT5 | 0.8426 | 0.8417 | 0.8426 | 0.8390 | 0.8060 |
|  | ProtPhage-Blosum64 | 0.6186 | 0.6380 | 0.6186 | 0.6227 | 0.5349 |
|  | ProtPhage-Physicochemical | 0.3313 | 0.2537 | 0.3313 | 0.2853 | 0.1447 |
| 90 | ProtPhage-ProtT5 | 0.8707 | 0.8713 | 0.8707 | 0.8678 | 0.8407 |
|  | ProtPhage-Blosum64 | 0.6508 | 0.6249 | 0.6508 | 0.6263 | 0.5654 |
|  | ProtPhage-Physicochemical | 0.3118 | 0.2655 | 0.3118 | 0.2729 | 0.1439 |

1. PVP function annotations

**Table S2.** PDRPxv genome PVP classification by ProtPhage

| Protein ID | Putative functiona | ProtPhage prediction | Confidence scores |
| --- | --- | --- | --- |
| Gp8 | Portal protein | Portal | 0.9989 |
| **Gp10** | **Minor head protein** | **Portal** | **0.6917** |
| **Gp11** | **Scaffolding protein** | **Baseplate** | **0.5716** |
| Gp12 | Major capsid protein | Major capsid | 0.9998 |
| Gp18 | Major tail subunit | Major tail | 0.9975 |
| **Gp25** | **Tail assembly chaperone** | **Minor tail** | **0.5616** |
| **Gp28** | **Tape measure protein** | **Tail fiber** | **0.3255** |
| Gp29 | Minor tail protein | Minor tail | 0.9999 |
| Gp30 | Minor tail protein | Minor tail | 0.9998 |
| Gp31 | Minor tail protein | Minor tail | 0.9995 |
| Gp32 | Minor tail protein | Minor tail | 0.9995 |
| Gp33 | Minor tail protein | Minor tail | 0.9989 |

**Bold** indicates the four sequences that were not correctly annotated.

1. Putative function as analysed by Sinha et al.[1]

**References**

1 Sinha, A., Eniyan, K., Manohar, P., Ramesh, N. & Bajpai, U. Characterization and genome analysis of B1 sub-cluster mycobacteriophage PDRPxv. Virus research 279, 197884 (2020).
